# Making Practical Markets for Vaccines: Why I decided that the Center for Global Development Report, *Making Markets for Vaccines*, offers poor advice to government and foundation leaders

**DOI:** 10.1371/journal.pmed.0020271

**Published:** 2005-09-13

**Authors:** Donald W Light

## Abstract

Donald Light raises concerns over the current proposal to fund vaccine development through advanced market commitments to companies.

This is the first of two articles in the October 2005 issue on ways to create practical markets for vaccines.

## 

“In the time it takes you to read this preface, 100 people will die of diseases that can already be prevented with vaccines, and 150 more will die of malaria, HIV or tuberculosis [1].” So begins Making Markets for Vaccines, a report from the Center for Global Development (CGD) in Washington, D.C. that is being vigorously promoted to leaders of the G8 and foundations as a blueprint for how to spend billions of dollars in donations to end the economic and personal burdens of so much suffering and loss.

In order to stop children “dying at the rate of an average-sized high school every hour,” the report offers a plan that is “simple and practical”—make an “advanced market commitment” (AMC) to purchase the equivalent of the revenues that the big multinational firms would receive from major Western drugs, once the vaccine or vaccines are discovered, tested, and brought to market. This commitment, says the report, will induce companies to start investing serious research funds and unleash the creative powers of their large research teams to discover new, effective vaccines. In return for a large buyout of a few billion dollars, the company or companies that win the windfall contract(s) would commit thereafter to sell all doses at a very low, cost-plus price (i.e., basic cost plus a small profit margin). Contracts would also depend on poor countries participating, paying a co-payment of about a dollar a dose, and meeting other requirements. The result will be that hundreds of millions of children will get immunized against deadly diseases so that they can learn, create, and unleash the productive potential in poor nations to transform themselves.[Fig pmed-0020271-g001]

**Figure pmed-0020271-g001:**
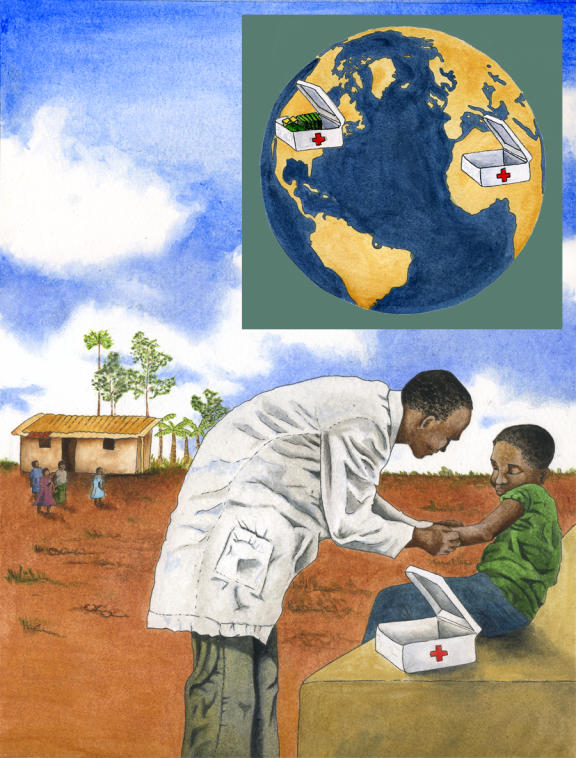
Vaccines won't reach their targets (Illustration: Emma Burns)

The AMC, which is not like a market in most ways, becomes a long-term contract best aimed at late-stage and existing vaccines, not at research for nonexistent vaccines. An advanced commitment is a slower, less efficient way to incentivize research to discover an effective new vaccine than direct research support. As a complement to public and charitable funding of research and development, an advanced commitment can buy many more million doses, to save millions more lives, at a much lower price because the risk and cost of research and development are being borne by the funders. While advanced commitments are a good idea for overcoming long delays due to patent enforcements, what leaders need is a different kind of report on how to make a big splash and a real difference with between US$1 billion and $5 billion, a report that outlines how advanced commitments can be most effective in saving lives and how the key issues in the manufacturing, organization, and delivery of vaccines in poor regions can be addressed. I will first identify the problems in what could be called the “core draft” of the CGD model by Michael Kremer, who holds the Gates professorship at Harvard, and then comment briefly on how the final report to leaders fudges and qualifies Kremer's model with add-ons to please all readers, so that what is being proposed becomes less coherent and more difficult to pin down.

## The Context

As a professor of comparative health-care systems, I served on the “Pull” Mechanisms Working Group (the Group) that the Gates Foundation funded the CGD to manage. Over the past several years, the Gates Foundation has transformed vaccine and drug research and development (R&D) for global diseases through bold funding and institution-building. These are called “push” efforts, because they use the direct force of contracts, funds, and grants to push along leading projects and programs. As a result, over a dozen promising new vaccines are entering clinical trials, or soon will be. But Kremer had proposed using the “pull” of a large financial commitment as the way to induce R&D in the private sector for neglected diseases (parts 1 and 2 of [2]). Though this is called an “advanced market commitment,” it is not a market, but one or a few donors making a large purchase. The Group should have explored and assessed the pros and cons of various pull mechanisms, but I felt it increasingly became a cheering squad for Kremer's model, which then was applied to malaria as the way to supersede the many previous efforts by government- and foundation-sponsored scientists to discover an effective vaccine. The more I learned as a neophyte about how weak the evidence was that this appealing idea would work and the ways it might make things worse, the more doubtful I became.

## Little Bang for Big Bucks

Two studies were featured in the report as proof that advanced commitments are a revolutionary technique to launch a new era of innovation. The study by Finkelstein provides a systematic analysis of how advanced commitment funding for vaccines has affected investments in R&D [3]. Finkelstein finds that only large firms respond to the inducement, by taking an already-discovered vaccine off the shelf and testing it, such as GlaxoSmithKline's (GSK's) vaccine candidate for malaria with modest, short-term efficacy [4]. Ironically, this is the only candidate mentioned in the Group's report, *Making Markets*…—yet it was push funding for testing, not pull inducement, that apparently got GSK to take it off the shelf after 15 years, and start trials. Finkelstein found that small firms, where most innovation is taking place, begin to participate later in a sustained larger market, which this advanced commitment model is not designed to create. Finkelstein concludes from her large sample that “for every $1 permanent increase in expected annual market revenue from vaccines against a particular disease [the CGD design], the pharmaceutical industry will spend an additional 6 cents annually in present discounted value on R&D for vaccines against that disease” (p. 543 of [3]).

The other major study cited in the CGD report as evidence that a $3 billion advanced commitment would have long, deep pull back to basic research (rather than short, shallow pull to fund clinical testing) comes to the implausible conclusion that just a 1% increase in market size leads to a 4%–6% increase in new drugs [5]. Not a 4- to 6-fold increase in research funding (also implausible) but a 4- to 6-fold increase in actual new drugs! This miraculous conclusion is stated as if it were fact, when it is based on a highly artificial econometric model. The model assumes that all individuals live indefinitely, that there is only one firm at any one time with the best-practice technology, that anticipated future market size (not actual size) prompts more innovation over long periods, and that “new drugs” include all generics and all newly approved drugs, even though less than 15% of the latter are therapeutically superior to existing drugs [6,7]. Like Finkelstein, the authors sensibly note that “pharmaceutical companies may respond more to profit incentives at the later stages of the research process than at the earlier stages.” Thus, both studies support using advanced commitments to encourage late-stage development, not basic research to discover new drugs or vaccines.

The CGD model creates a one-time market and does not address sustainability.

The studies cited to prove that donor-pull will spur companies to invest in basic research that might or might not discover an effective vaccine 10–15 years down the road in fact offer dubious evidence. Further, the vaccine business is technically different from drugs, and most of the big companies decided years ago to get out of it. Is an advanced commitment for one vaccine (or one disease—an ambiguity that creates further problems) enough to get them back into the vaccine business? A central problem is that the CGD model creates a one-time market and does not address sustainability. Meanwhile, the few companies that have vaccine research teams are already being funded directly or through public–private partnerships (PPPs), often by the Gates Foundation, so that an AMC for research is unnecessary. Finally, going after a big contract designed not to pay a penny until a company has invested a decade or more in discovery, development, testing, and approval is a less cost-effective way to commit billions of dollars than to do what Gates and others are doing already: funding the best basic research ideas (including from private-sector teams), creating PPPs and other bridging organizations, and bringing the best experts together in a global research community.

Market-induced basic research is still less plausible in the CGD model, because the more closely one reads the text, the less clear it becomes how much a company would actually get if it were to gamble hundreds of millions so that it could discover an effective vaccine. The core Kremer model comes up with $3 billion to match the average sales of an individual top-selling drug in order to make investing in research as attractive as for other products. But then it makes room for second or third successful vaccines by other companies, among whom the total amount of money has to be shared. Contracts also depend on the governments of each participating poor country agreeing to terms as subsidized purchasers. Then the final report shifts the argument from an advanced commitment for a vaccine to an advanced commitment for all vaccines for a given disease. In sum, these provisions make it unclear how much a company would get after years of R&D investments.[Table pmed-0020271-t001]

**Table 1 pmed-0020271-t001:**
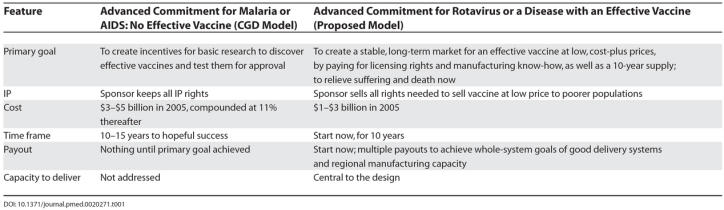
Contrasting Models of Advanced Purchase Commitments

These same provisions also make a binding contract impossible, because the donor cannot specify what it would pay a company if it invests in research to discover a new vaccine. And what is a company to make of the assurance that an advanced commitment will not cost the donor a penny until an effective vaccine meets the contractual criteria? If the advanced commitment requires no set-aside, why should investors and companies think it's real and not subject to executive or political change? If donors' financial commitment is real, why not save real lives by committing to make an effective vaccine available to the world's poor now, rather than possibly save hypothetical lives years from now?

## The Scientific Barriers to Vaccines

Besides weak evidence that a $3 billion advanced commitment would induce basic research, nothing is mentioned about the daunting scientific barriers to developing a vaccine for either malaria or HIV-AIDS. The Kremer model assumes that creating a large purchase will induce a solution; but scientists who have done the research say that the scientific obstacles may be insurmountable because the targets are multiple and evolving. This observation leads to a more serious weakness in a global competition for a big contract: it rewards scientific secrecy rather than sharing, whereas the cooperative push efforts in recent years have fostered partnerships and sharing. Here is a stark trade-off. Which is more likely to lead to better vaccines faster—fierce competition for a big future payoff or cooperative sponsorship and PPPs? The more cooperative government, university and nonprofit research teams will probably get nothing under the advanced commitment model.

The other big trade-off question was (and is): will committing large sums to the deep, long pull of an advanced commitment mean less money for grants and contracts to push vaccine development forward? The report asserts it would not. I find that suspect. I was told, in support of this assertion, that wealthy countries are ready to commit billions, and then billions more, to eradicating global diseases of the poor beyond the multinational scheme to buy and administer existing but underused vaccines. Is that true? If so, why have at least two studies concluded that foundations and governments (especially European) have not yet adequately funded R&D for neglected diseases [8,9]? Three billion dollars more for research will foster more innovation than $3 billion committed as an inducement for more research.

The big trade-off question gets buried by emphasizing that advanced commitments are to be added to current push efforts to “complement” them, as if committing a few billion dollars to “pull” funding has no effect on “push” funding. But if it does, the CGD report itself documents how much more progress has been made, for a fraction the cost, through directly funded grants and programs. Ironically, complementary uses of pull mechanisms with push ones were little discussed by the Group over the months of deliberation. Criticisms of the Kremer draft led to softening the final report but not to substantive development of synergistic combinations. Those are still waiting to be done.

To summarize, the rationale for Kremer's model, which still lies behind all the add-ons and qualifiers of the CGD final report, assumes that a large purchase will unleash innovative research to discover effective vaccines for the world's most intransigent diseases [10]. It is promoted, as Farlow notes of Kremer's book, “in much the same way that some pharmaceutical companies promote ‘;wonder drugs’: emphasizing the positives, burying the negatives, and ending up suggesting that we now have all the answers…” [11]. Neither evidence nor logic support the Kremer and CGD model, and advanced commitments for early-stage research can crowd out faster, more effective efforts both politically and economically. The CGD model belies its president's call for a “global commons” in which the best minds and teams work together for “a global social contract” to benefit humanity [1,7]. Why is there such a discrepancy between the rhetoric and the reality of the CGD model?

## Designed for Big Pharma

As drafts of the CGD report progressed, the number of contractual features and one-sided passages that favored the multinational corporations made me increasingly uncomfortable. Here are several examples:

Why is the advanced commitment contract designed so that competing firms get no money until a new vaccine is fully tested and approved? Only big Western firms have the cash reserves to sink hundreds of millions into research to discover and develop new vaccines, shutting out smaller companies in Asia, the Americas, and Africa. Interim and milestone payments were suggested but rejected as part of push grants, not pull AMCs. There are good reasons for using such payments in both initiatives. The final report keeps repeating that the process is open to all, but the contractual terms allow only cash-rich corporations to gamble for years for a possible big payoff and exclude future biotech companies that discover a vaccine after the initial contacts are signed.

Why do the winners get to keep patent rights, when these patent rights are the principal reason for the long delays in getting vaccines to poor countries at low prices? Drug companies with patent rights do not have a good record for sharing and building a global commons. Sharing and combining vaccines for malaria is especially important. A $3 billion advanced commitment is supposed to be a windfall buyout to shortcut access to poor nations, and it should include the rights and technical know-how needed for flexible capacity-building for that price. In fact, in many cases those rights could probably be bought for a tenth of that price.

What happened to the early goal of building up technical and manufacturing capacity in each continent? Several design features of the CGD model mitigate against it.

Why were principal legal advisers to big pharma chosen to do all the legal work, rather than a more neutral source? They are now coauthors with Group members as part of the promotional push for the “one true answer.” And why are the contractual term sheets drawn up by these advisers so vague in all the critical places? Innovative firms in Korea, India, China, Cuba, Brazil, or elsewhere outside the big pharma US–UK club are unlikely to trust this contractual process. Finally, why launch the report in the offices of principal legal advisers to big pharma?

Why is the cost of an advanced commitment set to the sales curves of drugs rather than to the sales curves of better-selling vaccines? Why does the report draw almost exclusively on industry-supported data and studies for the “facts” on which the advanced commitment is based? The result, when combined with the other points, is a bonanza for big pharma, and the text indicates that $3 billion is only a starting price, which is likely to increase rapidly to between $5 billion and $8 billion.

After the big payoff for 200 million courses, little is said (or was discussed) about how to sustain the vaccine effort. Sustainability is a major issue in vaccines for the poor; yet all the focus here appeared to be on a multi-billion dollar payment to big pharma.

Almost no time was spent analyzing the organizational, regulatory, and financial causes of past delays in making new vaccines available in poor countries. Will a $3 billion buyout solve all the sources of delay? Learning from the past did not seem to be the point.

Likewise, no time was spent understanding the organizational, political, and cultural barriers to effective delivery of the vaccines, only purchasing them. Rather than actually delivering vaccines to people, is a windfall purchase the real goal here? As an expert in health-care delivery, I could not endorse a report that ignored these issues.

Why is GSK's marginally effective vaccine candidate mentioned by name in the report—and why are the terms of contract then made loose enough so that a small, hand-picked committee is permitted to lower (but not raise!) the minimal thresholds for a vaccine to be acceptable?

Answers to such questions were brought into focus by the comment in Europe of a senior, international expert on vaccines and their markets. He explained that the major companies are running out of markets to sustain their rapid growth. That's why they're turning sexual performance or shyness into medical problems. They have been looking for years for a way to make a profitable market out of global vaccines, and in the CGD group's proposal it looks as if they have found a way: “Why don't they just say they want to give GSK $3 billion for their marginally effective vaccine?”

Were members of the CGD group being used as agents for this agenda?

## Making Markets for Sustainable Cheap Vaccines

The reasonable doubts here that led me to withhold my endorsement of the CGD report do not address a number of other serious concerns: how difficult, for instance, it is to get the buyout price, and especially the post-buyout price, right years in advance (Box 1). There are also problems with the contracts, the oversight committee, and liability issues; problems of inequities; and problems with the increasingly confused terms of what is being proposed—issues taken up in more detail elsewhere [12–14]. The G8 finance ministers have been misadvised to write that advanced purchase commitments are a potentially powerful mechanism to incentivize research [15]. But none of these problems detracts from my thinking that advanced purchase commitments are a good idea when applied where they work best: on existing vaccines that could save millions from suffering and dying now. It seems morally dubious for a foundation or nation to do otherwise. The singular omission in the Grand Challenges in Global Health is that they do not call for the eradication of all the diseases for which effective vaccines already exist [16,17]. When millions of lives could be saved now, why give priority to future lives that might or might not be saved?

**Box 1. The Price Is Wrong**

Remember the quiz show, “The Price is Right,” where contestants guessed how much things actually cost? A big problem with advanced purchase or market commitments is that guessing both the payoff price and the post-payoff price is very difficult when attempted years before one even knows the kind of effective vaccine that will be discovered and how it is to be administered.

To be fair to competitors, the payoff price should be adjusted for how much R&D was paid for by governments and foundations, because some risk much less of their own money, net of tax subsidies, than others. The CGD price of $15 per course and $3 billion might be much too high, or too low, by 2015. And no adjusters are mentioned for external subsidies and other factors.

The permanent post-payoff cost of manufacturing most vaccines in volumes above 10 million units, according to information given to our Group, is very low, between one and five cents. But a new-age biologic vaccine might cost much more. The CGD price of $1 per course is quite high compared to many generic vaccines and for many poor governments. But then, it might not cover the costs of a technically expensive vaccine. Both prices can be right, however, if an advanced commitment can be made for an existing vaccine to eradicate a dread disease.

An advanced commitment as a complement to paying for R&D could be designed to establish a sustainable, long-term market for an effective vaccine to eradicate a global disease. With little risk or private investment to pay off, one could commit to 600 million doses for $3 billion rather than 200 million doses. The terms should build in financial support as well as expert help to strengthen the public health delivery systems of recipient nations and their capacity to build the new vaccine into their budgets and planning. The donor could announce honestly that it is eradicating a global scourge, instead of saying that it might do so ten years from now. Licenses for low-income markets as well as manufacturing know-how would be part of the deal, and favoring regional manufacturers would be a related goal. Through this kind of flexible, long-term contracting focused on delivery and capacity-building, an advanced commitment could create sustainable, whole-systems markets for new vaccines that current R&D efforts are pushing forward. This is one idea, but we need the kind of report I described at the beginning, which assesses this model along with other forms of advanced commitments and push–pull combinations.

## Abbreviations

AMC: advanced market commitment
CGD: Center for Global Development
the Group: the “Pull” Mechanisms Working Group of the Center for Global Development
GSK: GlaxoSmithKline
PPP: public–private partnership
R&D: research and development.
